# A phase 1/2 study combining gemcitabine, Pegintron and p53 SLP vaccine in patients with platinum-resistant ovarian cancer

**DOI:** 10.18632/oncotarget.4772

**Published:** 2015-08-14

**Authors:** Eveline M. Dijkgraaf, Saskia J.A.M. Santegoets, An K.L. Reyners, Renske Goedemans, Hans W. Nijman, Mariëtte I.E. van Poelgeest, Arien R. van Erkel, Vincent T.H.B.M. Smit, Toos A.H.H. Daemen, Jacobus J.M. van der Hoeven, Cornelis J.M. Melief, Marij J.P. Welters, Judith R. Kroep, Sjoerd H. van der Burg

**Affiliations:** ^1^ Department of Medical Oncology, Leiden University Medical Center, 2333 ZA, Leiden, The Netherlands; ^2^ Department of Clinical Oncology, University Medical Center Groningen, University of Groningen, 9713 GZ, Groningen, The Netherlands; ^3^ Department of Gynecologic Oncology, University Medical Center Groningen, University of Groningen, 9713 GZ, Groningen, The Netherlands; ^4^ Department of Gynecology, Leiden University Medical Center, 2333 ZA, Leiden, The Netherlands; ^5^ Department of Radiology, Leiden University Medical Center, 2333 ZA, Leiden, The Netherlands; ^6^ Department of Pathology, Leiden University Medical Center, 2333 ZA, Leiden, The Netherlands; ^7^ Department of Medical Microbiology, University Medical Center Groningen, University of Groningen, 9713 GZ, Groningen, The Netherlands; ^8^ Department of Immunohematology and Blood Transfusion, Leiden University Medical Center, 2333 ZA, Leiden, The Netherlands

**Keywords:** ovarian cancer, p53, immunotherapy, chemoresistance, tumor immunity

## Abstract

**Purpose:**

Preclinical tumor models show that chemotherapy has immune modulatory properties which can be exploited in the context of immunotherapy. The purpose of this study was to determine the feasibility and immunogenicity of combinations of such an immunomodulatory chemotherapeutic agent with immunotherapy, p53 synthetic long peptide (SLP) vaccine and Pegintron (IFN-α) in patients with platinum-resistant p53-positive epithelial ovarian cancer (EOC).

**Experimental design:**

This is a phase 1/2 trial in which patients sequential 6 cycles of gemcitabine (1000 mg/kg^2^ iv; *n* = 3), gemcitabine with Pegintron before and after the first gemcitabine cycle (Pegintron 1 μg/kg sc; *n* = 6), and gemcitabine and Pegintron combined with p53 SLP vaccine (0.3 mg/peptide, 9 peptides; *n* = 6). At baseline, 22 days after the 2^nd^ and 6^th^ cycle, blood was collected for immunomonitoring. Toxicity, CA-125, and radiologic response were evaluated after 3 and 6 cycles of chemotherapy.

**Results:**

None of the patients enrolled experienced dose-limiting toxicity. Predominant grade 3/4 toxicities were nausea/vomiting and dyspnea. Grade 1/2 toxicities consisted of fatigue (78%) and Pegintron-related flu-like symptoms (72%). Gemcitabine reduced myeloid-derived suppressor cells (*p* = 0.0005) and increased immune-supportive M1 macrophages (*p* = 0.04). Combination of gemcitabine and Pegintron stimulated higher frequencies of circulating proliferating CD4+ and CD8+ T-cells but not regulatory T-cells. All vaccinated patients showed strong vaccine-induced p53-specific T-cell responses.

**Conclusion:**

Combination of gemcitabine, the immune modulator Pegintron and therapeutic peptide vaccination is a viable approach in the development of combined chemo-immunotherapeutic regimens to treat cancer.

## INTRODUCTION

Ovarian cancer has a dismal prognosis, with a 5-years survival of 30% [1, 2]. These cancer patients are treated with platinum-based chemotherapy but the majority develops recurrences and ultimately die because of treatment failure, indicating that other treatment strategies are warranted. Cancer immunotherapy has been shown to be an effective treatment modality in metastatic cancer, however, clinical efficacy is often delayed and only observed in a part of the treated cancer patients [3], including those with ovarian cancer [4, 5]. Epithelial ovarian cancer (EOC) is likely to benefit from T-cell-based immunotherapy as it was observed that strong infiltration with CD8+ T-cells is correlated with enhanced survival and response to chemotherapy [6, 7]. Unfortunately, the EOC microenvironment is known to restrain the cytotoxic activity of effector lymphocytes in a direct fashion [8, 9] as well as indirectly by favoring the accumulation of regulatory T-cells (Tregs), myeloid-derived suppressor cells (MDSC) and M2 macrophages [10–12], associated with suppression of tumor immunity and treatment failures [13]. The path to clinical success in patients with EOC thus requires a strategy that increases the frequency and activity of antitumor T-cells while eliminating suppressive immune cells and providing enough time for a fully developed antitumor response, hence treatment should start as early as possible.

In the last decade, platinum-resistant EOC patients are often treated with gemcitabine as it has single agent activity and a favorable safety profile [14]. Interestingly, gemcitabine not only has direct anti-tumor effects but has also been shown to eliminate MDSCs and Tregs in preclinical tumor models [15–18]. In addition, we showed that gemcitabine delayed tumor growth and synergized with therapeutic vaccination for the eradication of established tumors in a murine tumor model [19]. Altogether, in preclinical studies gemcitabine could not only be successfully combined with therapeutic vaccination but it also deleted two types of immune suppressive cells playing a role in EOC.

Based on these preclinical data we hypothesized whether it was possible to combine gemcitabine with therapeutic vaccination and interferon alpha (IFN-α) in patients. In this study, we chose to use the p53 Synthetic Long Peptide vaccine (P53 SLP) to strengthen the tumor-specific immune response [20]. The p53 protein is overexpressed in about half of the ovarian cancer patients and known to activate spontaneous T-cell responses in these patients [21]. Previously, administration of the p53 SLP vaccine in patients with ovarian cancer was feasible, safe and showed to induce p53-specific T-cell responses [22]. Treatment with a low dose of cyclophosphamide to temporary decrease the number of Tregs, given before p53 SLP vaccination further increased the p53-specific immune responses, but did not improve clinical responses [23]. In colorectal cancer patients the combination of this p53 SLP vaccine with IFN-α on the injection site resulted in enhanced inflammation as well as stronger and better type 1 cytokine polarized p53-specific CD4+ and CD8+ T-cell responses [24]. IFN-α is known to induce the full maturation of dendritic cells, to improve cross-presentation of tumor antigens and to enhance survival of activated T-cells, thereby enhancing the anti-tumor response [25–30]. In preclinical mouse experiments, no tolerance of CD4+ T-cells to wt p53 was demonstrable [31, 32] and such CD4+ T-cells enhanced the anti-tumor effect of tumor-specific CD8+ T-cells [31].

Here, we studied the feasibility and immunogenicity to treat patients with platinum-resistant p53-positive ovarian cancer using combinatorial regimens in which p53 SLP vaccination and Pegintron (IFN-α) were administrated before and after the first cycle of gemcitabine. Analysis of the effect of these compounds on the patient's immune system revealed a reduction in MDSC, an increase in both M1 macrophages and activated T-cells, as well as a strong reactivity against the p53 SLP vaccine.

## MATERIALS AND METHODS

### Patients

Patients were eligible for inclusion in the study if they were at least 18 years of age, had platinum-resistant ovarian cancer with immunohistochemically confirmed ‘mutant’ p53-expression pattern, defined as a strong nuclear staining in more than 75% of the tumor cells, had measurable disease (RECIST 1.1) or elevated CA-125 > 2 times the upper limit. Patients also had to have a WHO performance score of 0–2 and adequate bone marrow function (WBC ≥3.0 × 10^9^/L, neutrophils ≥ 1.5 × 10^9^/L, platelets ≥100 × 10^9^/L), liver function (bilirubin ≤1.5 × upper limit of normal (UNL) range, ALAT and/or ASAT ≤2.5 × UNL, Alkaline Phosphatase ≤5 × UNL) and renal function (calculated creatinine clearance ≥50 mL/min). Written informed consent was obtained from all patients. Patients were excluded from the study when they had a malignancy within the previous 5 years (with exception of a history of a previous basal cell carcinoma of the skin or pre-invasive carcinoma of the cervix), serious other diseases, known hypersensitivity to any of the components of the treatment, were pregnant or lactating or had any medical or psychological condition which in the opinion of the investigator would not permit the patient to complete the study or sign meaningful informed consent. The study was ethically approved by the Central Committee on Research Involving Human Subjects in The Hague, The Netherlands (NL34041.000.10) and registered at clinicaltrials.gov (NTC01639885).

### Study objectives

Primary objective was to determine the feasibility and immunogenicity of the combination of gemcitabine and interferon alpha-2b with or without p53 SLP. To assess the primary endpoint of feasibility, the incidence and severity of all adverse events, vital parameters and changes in blood chemistry and hematology parameters were determined. Toxicity was measured using the Common Terminology Criteria for Adverse Events v4.03 (CTCAEv4.03). Relationship to treatment was evaluated for all adverse events. At each visit, patients were assessed by physical examination, vital signs, toxicity and complete blood count with differential and serum biochemistry. The immunogenicity was determined by assessment of the induction of p53-specific T-cells following treatment. Secondary endpoints were assessment of the effect of chemo-immunotherapy on the immune system and the relationship between anti-tumor immunity and clinical outcome. The effect on the immune system was measured by an array of immunologic assays as described below (see: *immunomonitoring*). Tumor response to treatment was evaluated according to gynecological cancer intergroup (GCIG) criteria [33] by combining serum CA-125 levels obtained at every visit with computerized tomography (CT) performed within three weeks after third and sixth cycle of chemotherapy and evaluated according to RECIST criteria 1.1 [34].

### Treatment schedule

**Figure 1:**
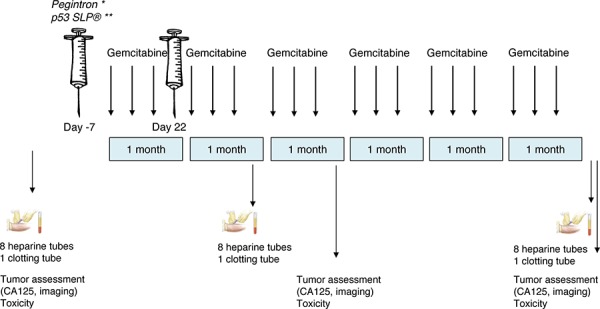
Study scheme This study consisted of three treatment groups: 1. 3 patients receiving only gemcitabine; 2. 6 patients receiving gemcitabine and Pegintron; 3. 6 patients receiving gemcitabine, Pegintron and p53 SLP. Before, after 2 cycles and after the last cycle, blood was drawn for immunomonitoring. Tumor assessment was performed at baseline, after 3 and after 6 cycles. *Pegintron was given 7 days prior to the first dose of gemcitabine and day 22 (Group 2). **The combination Pegintron and p53 SLP^®^ 7 days prior to the first dose of gemcitabine and day 22 (Group 3).

This was an open-label, multi-center, sequential trial. All patients received standard chemotherapy, 6 cycles of gemcitabine 1000 mg/m^2^ iv (d1, 8, 15; every 4 weeks). Patients were sequentially treated in three groups: the first three patients received gemcitabine alone, the following six patients received gemcitabine and IFN-α 2b s.c. (Pegintron 1 μg/kg, Schering-Plough, The Netherlands) 7 days prior and 22 days after to the first infusion of gemcitabine. The last cohort of six patients received gemcitabine, Pegintron and additionally p53 SLP vaccine (0.3 mg.peptide) in the same treatment schedule (Figure 1). The Pegintron as well as the vaccination were injected in the upper arm; Pegintron was injected within 10 centimeters proximity to the vaccination site. At baseline, day 22 of second cycle gemcitabine and at the end of the study, blood was drawn for immune-monitoring. To evaluate the impact of treatment on the immune system at least 5 evaluable patients (defined by a blood sample taken before and after treatment) were required in every intervention group.

### Vaccine

The p53 SLP vaccine consisted of 9 synthetic 25–30 amino acids long overlapping peptides, spanning amino acids 70–235 of the wt-p53 protein. Peptides were prepared at the GMP facility of the Department of Clinical Pharmacy and Toxicology at the LUMC. At the day of immunization the peptides (0.3 mg/peptide) were dissolved in dimethyl sulfoxide (DMSO, final concentration 20%) admixed with 20 mM phosphate buffer (pH 7.5) and emulsified with an equal volume of Montanide ISA-51 (Seppic). At the day of vaccination, the vaccine was prepared as previously described [24]. The vaccine (2.7 mL) was administered subcutaneously in the upper arm.

### Expression of p53

The expression of p53 by ovarian tumor cells was determined in the available paraffin-embedded metastatic tissue of the vaccinated patients by standard two-step indirect immunohistochemical staining as described previously [20]. Strong nuclear expression of p53 in ≥75% of the tumor cells was considered positive.

### Immunomonitoring

We acknowledge the concept of the Minimal Information About T-cell Assays (MIATA) reporting framework for human T-cell assays [35].

#### Cell samples

Venous blood (80 cmL) samples were drawn prior to vaccination, three weeks after the second chemotherapy cycle and at the end of study (three weeks after the sixth cycle of chemotherapy or if previously stopped at end of study). Peripheral blood mononuclear cells (PBMCs) and blood serum were isolated and stored as described previously [24]. To compare patient's immune characteristics with healthy donors, blood of six age-matched healthy donors was collected, processed and stored identically.

#### Enzyme linked immunospot (ELISPOT) assay

PBMCs prior to and post vaccination were thawed at the same time and subjected to the assay as described previously [24]. Briefly, a set of six pools of long overlapping peptides, indicated by the first and last amino acid in the p53 protein, was used for the screening of T-cell responses: p53.1: 1–78; p53.2: 70–115; p53.3: 102–155; p53.4: 142–203; p53.5: 190–248; and p53.6: 241–393. Peptide pools p53.2 to p53.5 represented the sequence in the p53 protein included in the vaccine, whereas the other two peptide pools p53.1 and p53.6 represented the remaining and flanking parts of p53. As a positive control, recall antigen mixture, the memory response mix (MRM), was taken along. Memory Response Mix (MRM; stock 4 ×), consisting of tetanus toxoid (0.06 LF/mL; National Institute of Public Health and the Environment, Bilthoven, The Netherlands), mycobacterium tuberculosis sonicate (0.4 μg/mL; Royal Tropical Institute, Amsterdam, The Netherlands) and Candida Albicans (0.0012%; HAL Allergenen Lab, Haarlem, The Netherlands).

The plates were read by automated ELISPOT reader (BioSys, Karben, Germany) according to guidelines as published [36]. Specific spots (mean number of spots in the test wells minus the mean number of spots plus 2x standard deviation (STD) in medium only control wells) of at least 1 in 10,000 PBMCs is considered a positive antigen-specific T-cell response. A vaccine-induced response was defined as at least a 3-fold increase in response after vaccination compared with the baseline sample.

#### Analysis of lymphocyte proliferation assay

The proliferative capacity of T-cells to mitogenic stimulation was analyzed as described previously [20]. The mean plus 3x STD of 4 medium control wells was used as cut-off value. The stimulation index (SI) was calculated by dividing the mean of test wells by that of the control wells. A positive response was defined as SI ≥ 3.

#### Antigen presenting cell capacity

PBMCs were tested in a mixed lymphocyte reaction (MLR) to evaluate the antigen presenting capacity as described before [24].

#### Immunophenotyping by flow cytometry

The immune cell composition of PBMCs was analyzed by flow cytometry as described previously [37]. Gating strategies are shown in Supplementary Figure S1. In short, a million cells were spin down and afterwards, 1mL lysisbuffer was added for 1 minute. After addition of 9 mL IMDM + 10% fetal cow serum (FCS), cells were centrifuged and staining was performed in PBS/0·5% BSA. Fc-receptor was blocked 10 minutes on ice by adding 50 μl PBS/0·5% BSA/10% FCS. Cells were incubated for 30 minutes with a mixture of the following antibodies: CD1a (FITC, clone HI149 – BD, Breda, The Netherlands), CD3 (Pacific Blue, clone UCHT1 – DAKO, Heverlee, Belgium or V450, clone UCHT1 – BD), CD4 (Horizon V500, clone RPA-T4 – BD), CD8 (APC-Cy7, clone SK1 – BD), CD11b (PE, clone D12 – BD or Alexa Fluor (AF) 488, clone CBRM1/5 – Biolegend, Uithoorn, the Netherlands), CD11c (AF700, clone B-ly6 – BD), CD14 (FITC, clone M5E2 – BD or PE-Cy7, clone M5E2 – BD or AF700, clone M5E2 – BD), CD15 (PE CF594, clone W6D3 – BD), CD16 (PE CF594, clone 3G8 – BD), CD19 (Brilliant Voilet (BV) 605, clone SJ25C1 – BD), CD33 (AF700, clone WM53 – BD or PE-Cy7, cloneP67·7 – BD), CD34 APC, clone 581 – BD), CD45 (PerCP-Cy5·5, clone 2D1 – BD), CD 56 (APC-Cy7 – Biolegend), CD124 (IL-4R; PE, clone HiL4R-M57 – BD), CD126 (IL-6R; PE, clone M5 – BD), CD163 (APC, clone 215927 – R&D, Systems, Minneapolis, MN), CD206 (Mannose Receptor; APC-Cy7, clone 15–2 – Biolegend), LIVE-DEAD^®^ Fixable yellow dead cell stain kit (Q-dot585 – Life technologies, Oregon, USA), HLA-DR (V500, clone L243 – BD), pSTAT1 (PE, clone py701 – BD), pSTAT3 (AF647, clone 49 – BD), pSTAT5 (PE, clone pY694 – BD), pSTAT6 (AF648, clone 18 – BD). The data were acquired on a the Fortessa (BD) and analysed with DIVA software version 6.2 and FlowJo version 7.0.

For the detection of Tregs 1 million PBMC were used per condition. Cell surface antibody staining of PBMC was performed in PBS/0·5%/BSA/0·02% sodium-azide (PBA) buffer for 30 minutes at 4°C. Intracytoplasmic/intranuclear staining was conducted with the BD Pharmingen Transcription Factor Buffer set (BD) according to manufacturers’ protocol. The antibodies used are: CD3 (V500, clone UCHT1 – BD), CD4 (AF700, clone RPA-T4 – BD), CD25 (PE-CY7, clone 2A3 – BD), CD127 (BV650, clone HIL-7R-M21 – BD), CD45RA (APC-H7, clone HI100 – BD), CD8 (PerCPCy5·5, clone SK1 – BD), FoxP3 (PE-CF594, clone 256D/C7 – BD), CTLA-4 (BV421, clone BNI3 – BD), Ki67 (FITC, clone 20Raj1 – eBiosciences, Vienna, Austria), Helios (APC, clone 22F6 – Biolegend) and LIVE-DEAD^®^ Fixable yellow dead cell stain kit (Q-dot585). The data were acquired on a the Fortessa (BD) and analysed with DIVA software version 6.2.

#### Determination of cytokines

IL-1β, IL-8, IL-10, TNF-α, IL-12p70 Inflammatory cytometric bead array (CBA, BD Biosciences) was used to determine cytokines and concentration (in pg/mL) present in supernatant of above described assays according to the manufacturer's instructions [32].

#### Laboratory environment

The immunomonitoring assays were performed in the laboratory of the department of Medical Oncology (LUMC) that operates under research conditions, externally and internally audited with respect to immunomonitoring, following SOPs, with pre-established definitions of positive responses and using trained staff. This laboratory has participated in all proficiency panels of the CIMT Immunoguiding Program (CIP; of which SHvdB and MW are steering committee members; http://www.cimt.eu/workgroups/cip/) to validate its SOPs as well as many of the proficiency panels of the USA-based Cancer Immunotherapy Consortium (CIC of the Cancer Research Institute).

### Statistical analysis

Based on our previous studies [20, 22], a sample size of six patients in each intervention group was sufficient to measure p53-specific reactions and the effect on Pegintron. No further power calculation was performed, since this was an exploratory study. For each individual immune-modulatory assay, a positive response is predefined per assay and described previously. Statistical analyses were conducted in SPSS (version 20 for Windows; SPSS, Inc). The Mann-Whitney test and the Fisher's exact test were used to evaluate differences in patient characteristics at different time points. The relationship between anti-tumor immunity and clinical outcome was determined using *t*-test and correlation tests (Pearson/Spearman). Survival curves were calculated using Kaplan-Meier method. Because this is a dose-finding, hypothesizing generating study, the data is not corrected for multiple comparisons.

## RESULTS

### Patient characteristics

Eighteen patients (median age 61 years, range 51–69) were enrolled between January 2010 and March 2013 in two Dutch hospitals (Leiden University medical Center (LUMC) and University Medical Center Groningen (UMCG). Patient characteristics are shown in Table 1. The mean time from diagnosis until inclusion was 31.4 months with a median of 2 (range 1–5) previous chemotherapy lines. Five patients ended the study before the second blood acquisition was achieved due to progression of disease. To determine the impact of treatment on the immune system in every therapy group, three more patients (C16, C17 and C18) were enrolled in the second group (gemcitabine + Pegintron). One patient (C08) received only one injection of Pegintron because of adverse events (flu-like symptoms grade 2) and one patient (C12) received only one vaccination and Pegintron injection, due to adverse events (redness of arm >10 cm and flu-like symptoms grade 2; Table 2).

**Table 1:** Patient characteristics and outcome

ID	Age	WHO	FIGO stage	Histology	Time	Current treatment	Treatment				Clinical response		Survival (weeks)	
							Cycles gemcitabine	Dose	p53 SLP^®^	Pegintron	Radiology results	CA125	PFS	OS
**Gemcitabine**														
**C01**	65	1	IIIc	Sereus	14	Third line	3		n	n	PD	PD	9	15
***C02***	*55*	*2*	*IV*	*Sereus*	*18*	*Third line*	*1*	*75% (1)*	*n*	*n*	*PD*	*na*	*5*	*5*
**C03**	53	1	IV	Sereus	14	Third line	6	75% (3)	n	n	SD	PD	16	73
**Gemcitabine + Pegintron**														
***C04***	*59*	*1*	*IIIc*	*Sereus*	*13*	*Second line*	*1*		*n*	*y*^‡^	*na*	*na*	*4*	*9*
***C05***	*68*	*1*	*IIIc*	*Sereus*	*4*	*Second line*	*2*		*n*	*y*	*na*	*na*	*12*	*17*
**C06**	67	0	III	Sereus	63	Sixth line	3		n	y	PD	SD	10	11
***C07***	*55*	*1*	*IV*	*Sereus*	*22*	*Third line*	*2*		*n*	*y*	*PD*	*na*	*6*	*7*
**C08**	51	0	IIIc	Endome trioid	44	Fourth line	2	60% (1)	n	y^‡^	PD	SD	13	23
***C09***	*65*	*0*	*III*	*Sereus*	*30*	*Fifth line*	*1*		*n*	*y*	*PD*	*na*	*7*	*8*
**C16**	58	1	IIIc	Sereus	22	Third line	3	75% (1)	n	y	PD	PR	11	ongoing
**C17**	69	1	IIIc	Sereus	12	Second line	6	75% (1)	n	y	SD	CR	60	ongoing
**C18**	67	0	IIb	Sereus	38	Third line	2	75% (1)	n	y	SD	nd	13	58
**Gemcitabine + Pegintron + p53 SLP**^®^														
**C10**	57	1	IIIc	Sereus	22	Third line	3	75% (1)	y	y	PD	PD	13	39
**C11**	65	0	IIIc	Endome trioid	17	Third line	3		y	y	PD	PD	11	25
**C12**	58	1	IIIc	Sereus	11	Second line	3	50% (1)	y^¥^	y	PD	PD	11	37
**C13**	69	0	IV	Sereus	93	Third line	6		y	y	SD	PR	20	48
**C14**	57	1	IV	Sereus	107	Sixth line	6	80% (4)	y	y	PR	PR	36	ongoing
**C15**	58	1	IV	Sereus	21	Third line	2		y	y	PR	PD	8	12

Abbreviations: LUMC = Leiden University Medical Center; UMCG = University Medical Center Groningen; WHO = World Health Organisation; FIGO = International Federation of Gynecology and Obstetrics; CA: cancer antigen; CR; complete response; SD: stable disease; PR: partial response; PD: progressive disease; PFS: time from start therapy till progression of disease; OS: time form start therapy till death; na: not available; nd: not detectable WHO at inclusion; FIGO stage at diagnosis; Time defines time from diagnosis until inclusion (weeks)

Italic reflect patients with only a blood sample at baseline, and therefore not suitable for immunomonitoring

‡ patient received only 1 dose of Peg-Intron

¥ patient received only 1 vaccination

**Table 2:** Adverse events

A.	Adverse events	Gemcitabine *n* = 3	Gemcitabine + Pegintron *n* = 9	Gemcitabine + Pegintron + p53 SLP^®^ *n* = 6
	**Grade 3/4 Adverse Events**			
	Abdominal pain		1	
	Infection			1
	Nausea/Vomiting	1	3	
	Dyspnea	1	1	1
	**Grade 1/2 Adverse Events in > 15% of patients**			
	Fatigue	3	7	4
	Flu-like symptoms	1	7	5
	Nausea/Vomiting	2	6	4
	Constipation	1	5	3
	Diarrhea		3	1
	Anorexia	3	4	1
	Abdominal pain		2	1
	**Blood Chemistry Adverse Events Grade 3**			
	Hypokalemia	1		
	Hyperkalemia	1		

B.	Local adverse events	Gemcitabine + Pegintron		Gemcitabine + Pegintron + p53 SLP^®^	
	Injection sites	>1 week *n* = 11	>2 months *n* = 11	>1 week *n* = 11	>2 months *n* = 11
	**Swelling**	**0 (0%)**	**0 (0%)**	**11 (100%)**	**9 (82%)**
	<5 cm			4 (36%)	2 (18%)
	5-10cm			4 (36%)	4 (36%)
	>10cm			3 (27%)	3 (27%)
	**Erythema**	**0 (0%)**	**0 (0%)**	**11 (100%)**	**9 (82%)**
	mild			8 (73%)	8 (73%)
	moderate			3 (27%)	1 (9%)
	severe			0 (0%)	0 (0%)
	**Temperature**	**0 (0%)**	**0 (0%)**	**11 (100%)**	**0 (0%)**
	mild			2 (18%)	0 (0%)
	moderate			7 (64%)	0 (0%)
	severe			2 (18%)	0 (0%)
	**Pain**	**0 (0%)**	**0 (0%)**	**4 (36%)**	**2 (18%)**
	mild			4 (36%)	2 (18%)
	moderate			0 (0%)	0 (0%)
	severe			0 (0%)	0 (0%)
	**Itching**	**0 (0%)**	**0 (0%)**	**3 (27%)**	**2 (18%)**
	mild			3 (27%)	0 (0%)
	moderate			0 (0%)	0 (0%)
	severe			0 (0%)	0 (0%)
	**Ulceration**	**0 (0%)**	**0 (0%)**	**0 (0%)**	**0 (0%)**

A) Adverse events, subdivided in different treatment groups. All grade 3/4 adverse events, adverse events occurring in more than 15% of all patients and blood chemistry adverse events > grade 2 are shown.B) Local adverse events at injection site. Adverse events at injection site were scored every visit and are subdivided here in early (after 1 week) and late (after 2 months) effects.

### Safety and tolerability

#### Adverse events

All adverse events are depicted in Table 2A. Nine patients (50%) showed grade 3 or 4 adverse events (Table 2A). The most frequent grade 3 or 4 adverse events were nausea/vomiting (22%) and dyspnea (17%). Four patients were admitted to the hospital because of severe nausea and vomiting. One patient had severe abdominal pain (unknown cause) which resolved spontaneously. Three patients had severe dyspnea due to their disease of which one had progressive disease and two could continue treatment after drainage of pleural fluid. The non-neutropenia fever was due to a nephrodrain induced Klebsiella pneumonia. No significant changes between the different treatment groups were found. One patient of the control group presented with a severe hypokalemia; in the group of gemcitabine combined with Pegintron one patient suffered from a grade 3 hyperkalemia.

The majority of patients experienced fatigue during treatment (78%), flu-like symptoms (72%) and nausea/vomiting (67%). Flu-like symptoms were observed 24 hours after injection of Pegintron in 12 of 13 patients. All other systemic adverse events occurring in > 15% of all patients are summarized in Table 2A and did not significantly differ between the treatment groups. All patients who received the p53 SLP vaccine developed grade 1–2 local skin reactions with redness and induration at the injection sites (Table 2B). This toxicity was long-lasting because 82% of the vaccination sites still were swollen and red (grade 1–2; Example shown in Supplementary Figure S2) after 2 months.

### Clinical outcome

A median of three gemcitabine cycles was administered (range 1 to 6). Fourteen patients did not complete all 6 chemotherapy cycles due to progression of disease. Based on CT-scan, a partial response (PR) was observed in two patients, stable disease (SD) in four patients and progressive disease (PD) in 10 patients. Two patients did not have a second CT-scan due to clinical PD. Outcome established by CA-125 levels resulted in one complete remission, three PR, two SD and six patients with PD. In five patients, CA-125 was not available, in one patient CA-125 was not evaluable.

### Immunogenicity

#### Gemcitabine reduces MDSCs and increases M1, but not M2 macrophages

**Figure 2:**
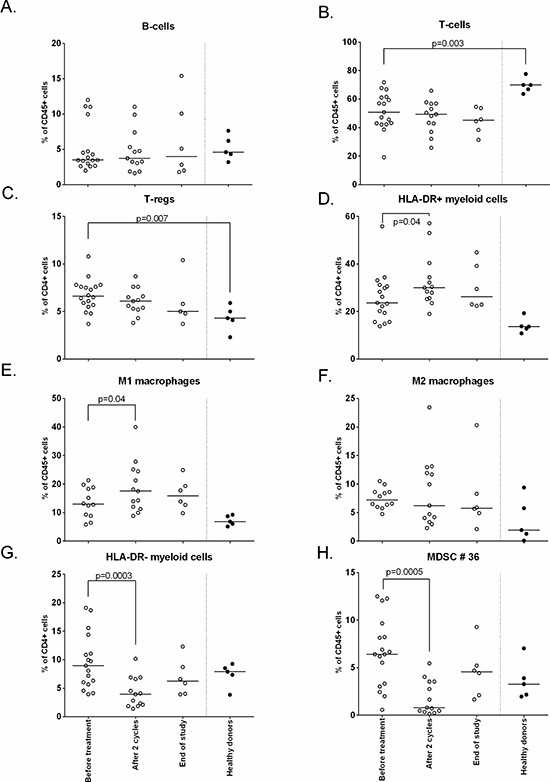
Phenotypical changes of different immune subsets upon treatment The immune cell composition was measured by flow cytometry at baseline, after 2 cycles and end of study. Depicted here are the following cell subsets: **A.** B-cells **B.** T-cells **C.** Tregs **D.** HLA-DR+ myeloid cells **E.** M1 macrophages **F.** M2 macrophages **G.** HLA-DR- myeloid cells H. MDSC #36. Treatment induces an increase in HLA-DR+ cells and M1 macrophages, HLA-DR- cells were decreased (*p* = 0.0003) and in particular myeloid cell population type 36 (*p* = 0.0005; defined as CD45+CD3−CD19−HLA−DR−CD11b+CD33−CD34−CD124−CD15−CD14−).

**Figure 3:**
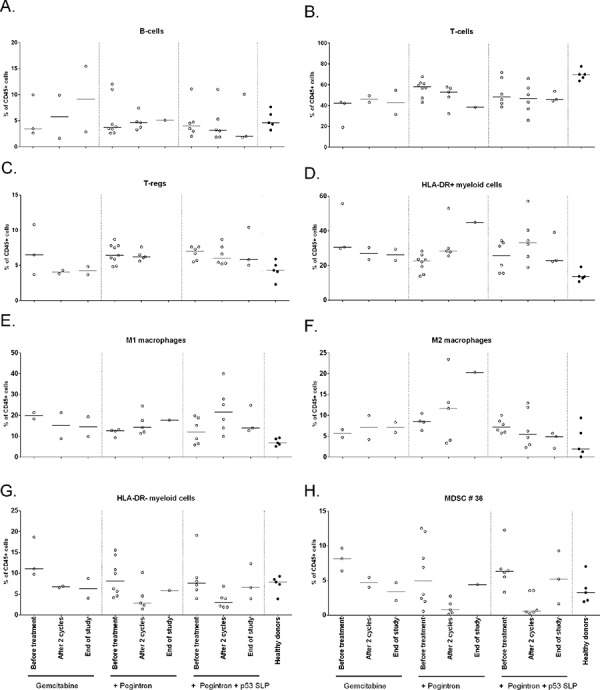
Phenotypical changes of different immune subsets upon treatment, divided per treatment group The immune cell composition as measured by flow cytometry is given for each of the three different treatment groups. **A.** B-cells **B.** T-cells **C.** Tregs **D.** HLA-DR+ myeloid cells **E.** M1 macrophages **F.** M2 macrophages **G.** HLA-DR- myeloid cells **H.** MDSC #36. In all treatment groups, including group 1 receiving only gemcitabine, HLA-DR- myeloid cells and MDSC #36 are decreased, suggesting that gemcitabine is responsible for the decrease of MDSCs after 2 cycles of chemotherapy.

In order to investigate the immunological effects of this triple treatment regimen, we studied the changes in phenotype of different immune cells. Although patients displayed lower frequencies of total number of T-cells and higher frequencies of CD25^pos^CD127^low^FoxP3^pos^ Tregs at baseline compared to healthy subjects, no changes in the frequencies of B cells, T-cells and Tregs were observed in response to the treatment. The total frequency of myeloid cells, defined by CD45+CD3-CD19- cells, was higher at baseline when compared to healthy donors but not affected by the treatment. Figure 2 shows the effect on all patients; Figure 3 shows the effect divided per treatment group. To get more insight in the effect of treatment on different subsets of the myeloid cell compartment, we analyzed the different subsets of MDSCs (HLA-DR- myeloid cells) and macrophages (HLA-DR+ myeloid cells). A classification of myeloid cell types based on our gating strategy (Supplementary Figure S1b) is shown in Supplementary Figure S3. Interestingly, the percentage of total HLA-DR + myeloid cells was increased upon treatment (*p* = 0.04; Figure 2D, Supplementary Figure S4), reflected by increases in CD11b+CD14+CD11c+CD163−CD16−CD206− macrophages (macrophage type 6; potentially M1 macrophages; *p* = 0.04), but not of CD11b+CD14+CD11c+CD163+CD16−CD206− macro phages (macrophage type 8; suppressive M2-like macrophages; Figure 2E, 2F, Supplementary Figure S4). Concomitantly, an explicit decline in HLA-DR- myeloid cells (*p* = 0.0003) was observed (Figure 2G, 2H, Supplementary Figure S4), in particular that of CD14−CD15−CD11b+CD33−CD34−CD124− (MDSC type 36; MDSC definition 10; *p* = 0.0005) in all treatment groups. The observed changes were found in all treatment groups (Figure 3, Supplementary Figure S5), suggesting that these effects are gemcitabine mediated.

#### Gemcitabine/Pegintron/p53 SLP treatment induces profound T-cell activation and increases in Activated T-cell/regulatory T cell ratios

**Figure 4:**
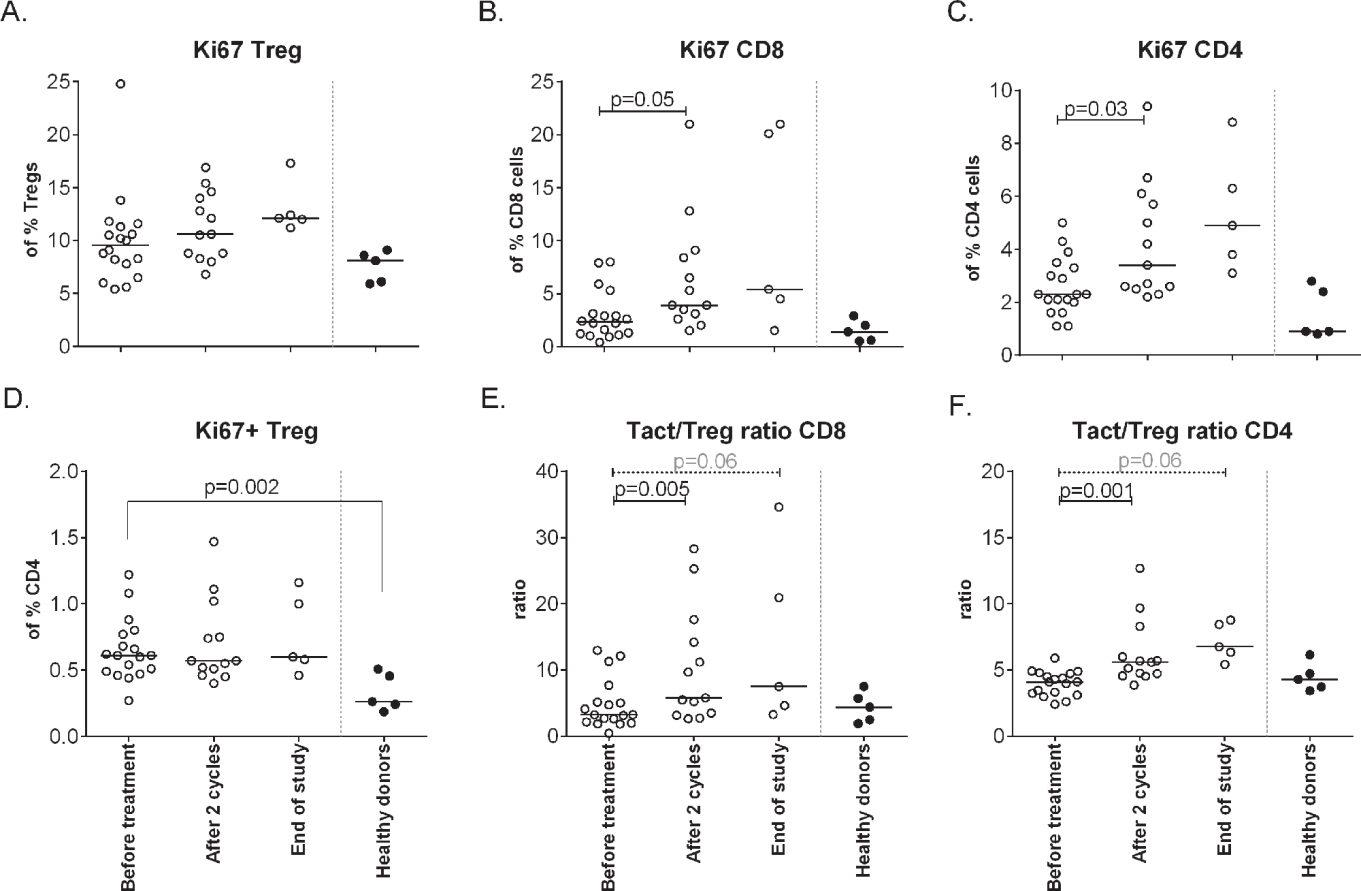
Changes in Ki67 expression on different cell subsets upon treatment Activation status of different T-cell subsets, as defined by Ki67+, was measured by flow cytometry at baseline, after 2 cycles and at end of study. **A.** The % of activated Tregs upon treatment. **B.** The percentage of activated CD8+ cells increases upon treatment (*p* = 0.05). **C.** Activated CD4+ T-cells are increased after therapy (*p* = 0.03). **D.** Activation status of CD4+ Tregsshowing less activated cells in patients compared to healthy donors. **E.** The ratio between activated CD8+ T-cells and activated Tregs increases upon treatment with the the balance in favor of activated CD8+ T-cells. **F.** The ratio between activated CD4+ T-cell and activated Tregs is in favor of the activated CD4+ T-cell.

**Figure 5:**
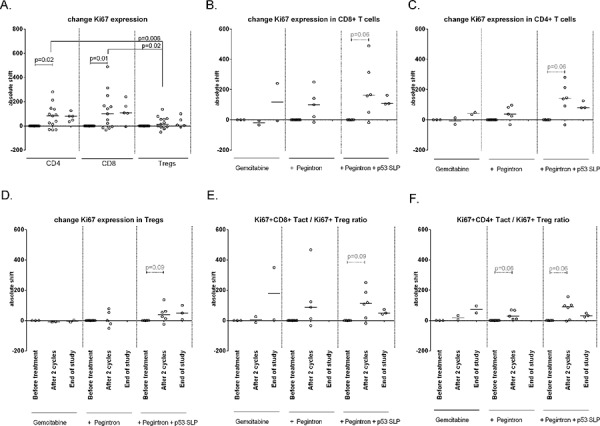
Changes in Ki67 expression on different cell subsets upon treatment, divided per treatment group Activation status of different T-cell subsets upon treatment, measured on baseline, after 2 cycles and at end of study. **A.** IIncreased – measured as an absolute shift – Ki67 expression in CD4+ (*p* = 0.02) and CD8+ cells (*p* = 0.01), but not on Tregs. **B-D.** Patients receiving gemcitabine, Pegintron and p53 SLP vaccination show increased Ki67 expression after 2 cycles of treatment by CD8+ T-cells (B), CD4+ T-cells (C) and by Tregs (D). **E–F.** The ratio between activated CD8+ or CD4+ T-cells and activated Tregs indicate a stronger increase in activated CD8+ cells (E), and non-Treges CD4+ T-cell (F).

In addition, the effect of treatment on the activation status of T-cells was studied. No changes in expression of CTLA-4 and CD45RA expression were observed in CD4+ and CD8+ T-cells and Tregs following treatment (not shown). Ki67 expression was detected in CD4+ Tregs, CD4+ non-Tregs and CD8+ T-cells, with Tregs displaying the highest percentages. Upon treatment Ki67 expression was significantly up-regulated in CD4+ and CD8+ T-cells but not in Tregs, resulting in increased CD8+Ki67+ and CD4+Ki67+ (activated T-cell) to Ki67+ (activated) Treg (Tact/Treg) ratios (Figure 4). Interestingly, these increases were observed whenever patients were treated with gemcitabine and IFN-α, irrespective of p53 vaccination (Figure 5).

#### Strength of immune response correlates with swelling of the injection site

**Figure 6:**
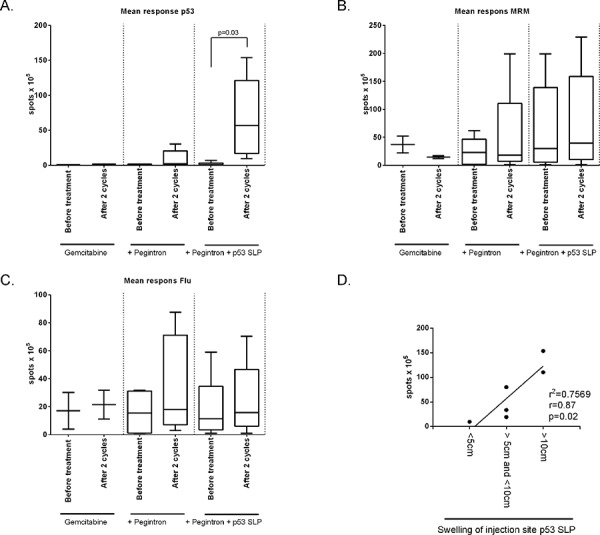
Strong p53-specific immune responses were measured after vaccination in combination with Pegintron Cellular immune responses to the vaccine were assessed by an IFN-γ ELISPOT assay. **A.** Mean response against p53 divided per study group. Vaccinated patients show a strong response after vaccination (*p* = 0.03). No changes were observed in the T-cell response to **B.** the recall antigen mix MRM or **C.** influenza M1 (Flu). **D.** Swelling of injection site correlates with strength of T-cell response measured by IFN-γ ELISPOT (*p* = 0.02), measured by linear regression (*r* = 0.87).

Cellular immune responses to the vaccine were assessed by an IFN-γ ELISPOT assay. At baseline, only one patient (C13) displayed a T-cell response to p53 peptide pool3. Control patients, receiving only chemotherapy, did not show a response to any of the p53 peptide pools in this assay. In the gemcitabine and Pegintron treatment group, two patients displayed a modest response after 2 cycles of treatment. P53-specific T-cell responses were detectable in all p53 SLP vaccinated patients after treatment (*p* = 0.03; Figure 6A, Supplementary Figure S6). The general T-cell response, i.e. the recall response to influenza virus M1 peptides as well as to a mix of bacterial antigens (MRM), showed a non-significant increase in reactivity in patients treated with gemcitabine and Pegintron, irrespective of p53 vaccination (Figure 6B). Interestingly, the magnitude of the swelling of the injection site was correlated with more IFN-γ producing p53-specific T-cells (*p* = 0.02; *r* = 0.87; Figure 6C).

#### Cytokine production and antigen presentation does not change upon treatment

The capacity of T-cells to proliferate upon antigenic stimulation was analyzed before and after treatment (Supplementary Figure S7A). Based on the amounts of cytokines secreted, the patients displayed a more pronounced Th1 profile, with high levels of IFN-γ and TNF-α rather than IL-4 or IL-5 (Supplementary Figure S7B). Neither the amount of cytokines produced nor the balance between the cytokines changed during treatment. In addition, there were no changes in the capacity of circulating antigen presenting cells to stimulate allogeneic lymphocyte reactions (Supplementary Figure S7C).

## DISCUSSION

Patients with platinum-resistant p53-positive ovarian cancer were treated with gemcitabine, gemcitabine with Pegintron, or a combination of gemcitabine, Pegintron and p53 SLP vaccine. The combination treatments were safe, feasible and had immune stimulatory effects. Our results show that gemcitabine treatment resulted in a reduction of immune-suppressive MDSCs and an increase in immune-stimulating M1 macrophages. Furthermore, the combination of gemcitabine and Pegintron stimulated higher frequencies of circulating proliferating T-cells but not Tregs. Moreover, all vaccinated patients showed a strong vaccine-induced p53-specific T-cell response.

We observed eleven grade 3/4 adverse events, most likely due to chemotherapy and/or Pegintron. To address the role of the combination of gemcitabine with Pegintron in bone marrow depletion would have required a group of patients treated with chemotherapy and vaccination only but such a group was not included because of the beneficial immunological effects of Pegintron on the T-cell response induced by the p53 SLP vaccine [24]. Pegintron-specific adverse events were flu-like symptoms reported within 24 hours after administration, but all were well manageable. Specific adverse events caused by the vaccine treatment were local redness and induration at the injection site which did not exceed grade 2 reactions. The observed skin reactions are in accordance with our previous study in which Pegintron was combined with the p53 SLP vaccine [24], and probably the result of a strong IFN-α potentiated (18–20, 27–29) immune response to the vaccine. This notion is strengthened by the explicit correlation between the size of the injection site (Figure 4D) and the response measured by ELISPOT (Figure 4A), as well as the presence of circulating Ki67+ T-cells in patients treated with gemcitabine and Pegintron (Figure 3).

Several mouse studies showed that gemcitabine is able to eliminate MDSCs and Tregs [15–18]. We are the first to show in humans that gemcitabine treatment decreases MDSC, in particular the CD11b+, HLA-DRlow (MDSC 10) population in humans (*p* = 0.0005). In addition, we showed that gemcitabine treatment resulted in an increase in M1 macrophages (*p* = 0.04) but importantly not in immune-suppressive M2 macrophages. Previously, it was shown that the population of circulating CD4+ Tregs displayed a higher proportion of ki67+ cells than non-Treg CD4+ T-cells or CD8+ T-cells [18]. Our study shows similar data on ki67 expression by T-cells. Of note, the small population study size makes the interpretation of the immunological data prone to false positive findings but it is encouraging to see that our data confirms the earlier findings in mouse models and patients Furthermore, it was shown that gemcitabine treatment depleted the majority of the Ki67+ cells, when measured 1–2 days after treatment, thereby disproportionally affecting Tregs [18]. We analyzed blood samples 7–14 days after gemcitabine treatment and although we did not observe a decrease in the frequency of Ki67 + T-cells at that time point, a significant increase in Ki67+ CD4+ and CD8+ T-cells was detected, which was not mirrored by the CD4 + Treg population. This resulted in increased Ki67 + T-cell over Ki67+ Treg ratio's, sustaining the notion that gemcitabine may affect Tregs more than other T-cells in humans even after the immediate effect of the drug has worn off.

Previous studies have shown that p53 SLP vaccination induced p53-specific T-cell responses in ovarian cancer patients [22] and that the combination with Pegintron resulted in stronger immune responses [24]. Here, the combination of Pegintron and p53 SLP resulted in a strong immune response reflected by the local vaccine site reactions and the T-cell response against the vaccine peptides as measured by IFN-γ ELISPOT assay. Compared to previous studies, concurrent administration of at least two cycles of gemcitabine does not affect p53-specific T-cell reactivity. Potentially this is also true for 6 cycles of gemcitabine but the number of patients tested at that point was too low for firm conclusions. It is important to emphasize that in the current study, there is no separate group combining p53 SLP vaccination with chemotherapy alone to study the influence of Pegintron on the p53-specific reactivity.

The combination of gemcitabine and IFN-α was shown to act synergistically in inhibiting tumor cell proliferation in a mouse model for pancreatic cancer [38, 39]. In a dose-finding phase 1 trial, Fuxius et al [40] combined gemcitabine at maximum tolerated dose (MTD) 1000 mg/m^2^ with IFN-α-2b 3 × weekly (MTD 5 × 10^6^ IU) for 3 consecutive weeks followed by 1 week of rest (28-day cycles) in patients with solid tumors, including one patient with ovarian cancer. This dose of IFNα is much higher than what we have used because our primary goal was to boost the immune response. The regimen was safe and associated with clinical data worth further investigations. Unfortunately, no immune monitoring was performed that would allow further comparison with our study.

This is a small dose finding study, and therefore underpowered to demonstrate efficacy in this challenging populationHowever, this study showed that gemcitabine treatment, immune modulation with Pegintron and therapeutic vaccination is a well-tolerated approach in the development of combined chemo-immunotherapeutic regimens to treat cancer. Gemcitabine may be administered as part of the standard of care, such as in this study, but can also be used in combination with T-cell stimulatory based immunotherapeutic strategies for its ability to decrease the number of immune suppressive in future studies.

## SUPPLEMENTARY FIGURES


