# Supplementary material for: A phase 1/2 study combining gemcitabine, Pegintron and p53 SLP vaccine in patients with platinum-resistant ovarian cancer
Source: Oncotarget. 2015 Aug 14;6(31):32228–43. doi: 10.18632/oncotarget.4772 (PMC4741673; doi:10.18632/oncotarget.4772)
Supplement: Supplementary file 1 [file oncotarget-06-32228-s001.pdf]

SUPPLEMENTARY FIGURES

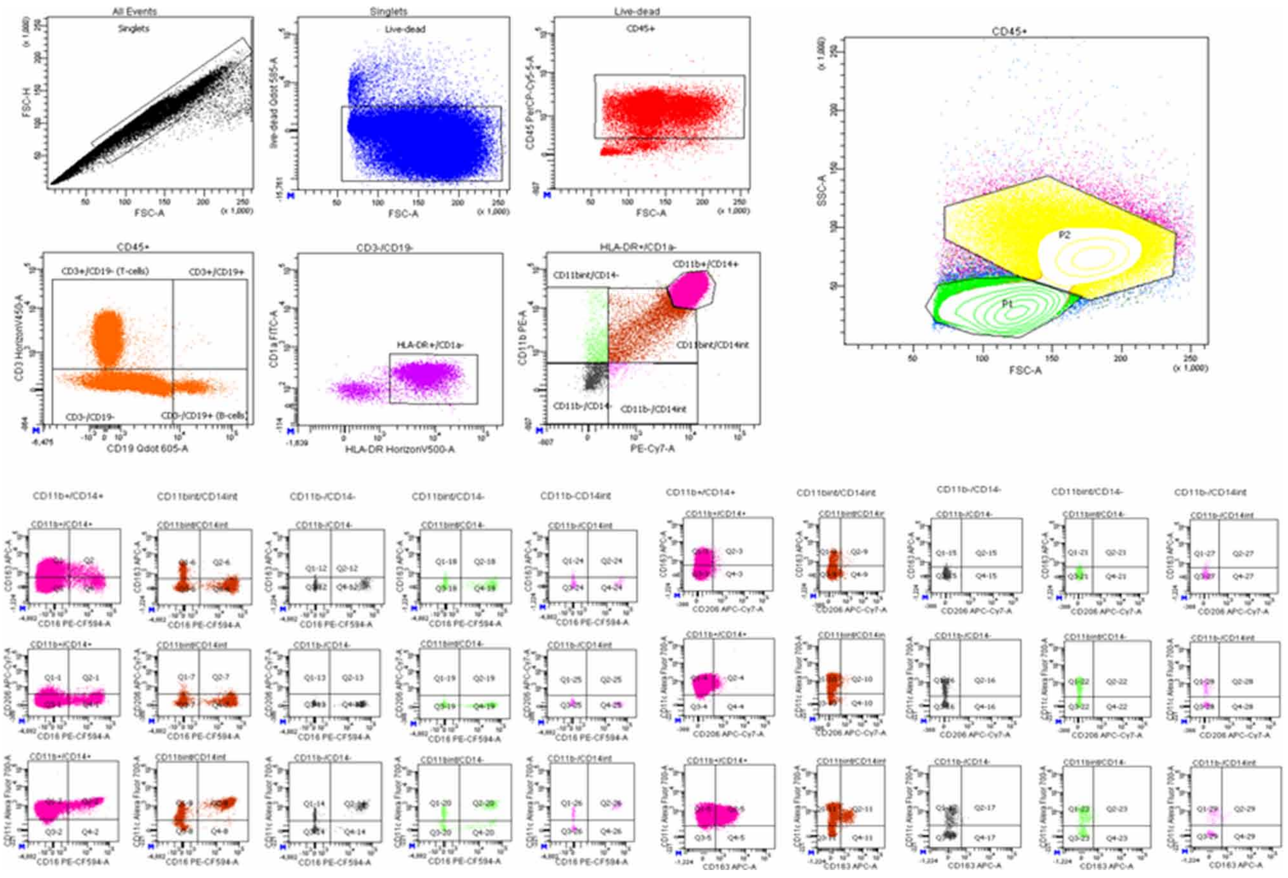

Supplementary Figure S1A: Gating strategy macrophages. Singlets were gated based on FCS-A/FCS-H properties, after which the dead cells were excluded through gating on Yellow-Fluorescent Reactive Dye (Y-ARD)-negative cells. Leukocytes were gated on FSC-A/CD45. Subsequently, T-cells (CD3+CD19−), B-cells (CD3-CD19+) and myeloid cells (CD3-CD19−) were gated on differential expression of CD3 and CD19. Myeloid cells were further divided into HLA-DR$_{pos}$/CD1a$_{neg}$ cells. Different macrophage subsets were split defined on CD14 and CD11b staining, resulting in 5 subcategories: CD11b+CD14+, CD11b$_{int}$CD14$_{int}$, CD11b−CD14−, CD11b$_{int}$CD14− and CD11b−CD14$_{int.}$ These subsets were then further subdivided based on CD163, CD16, CD206 and CD11c. (*Continued*)

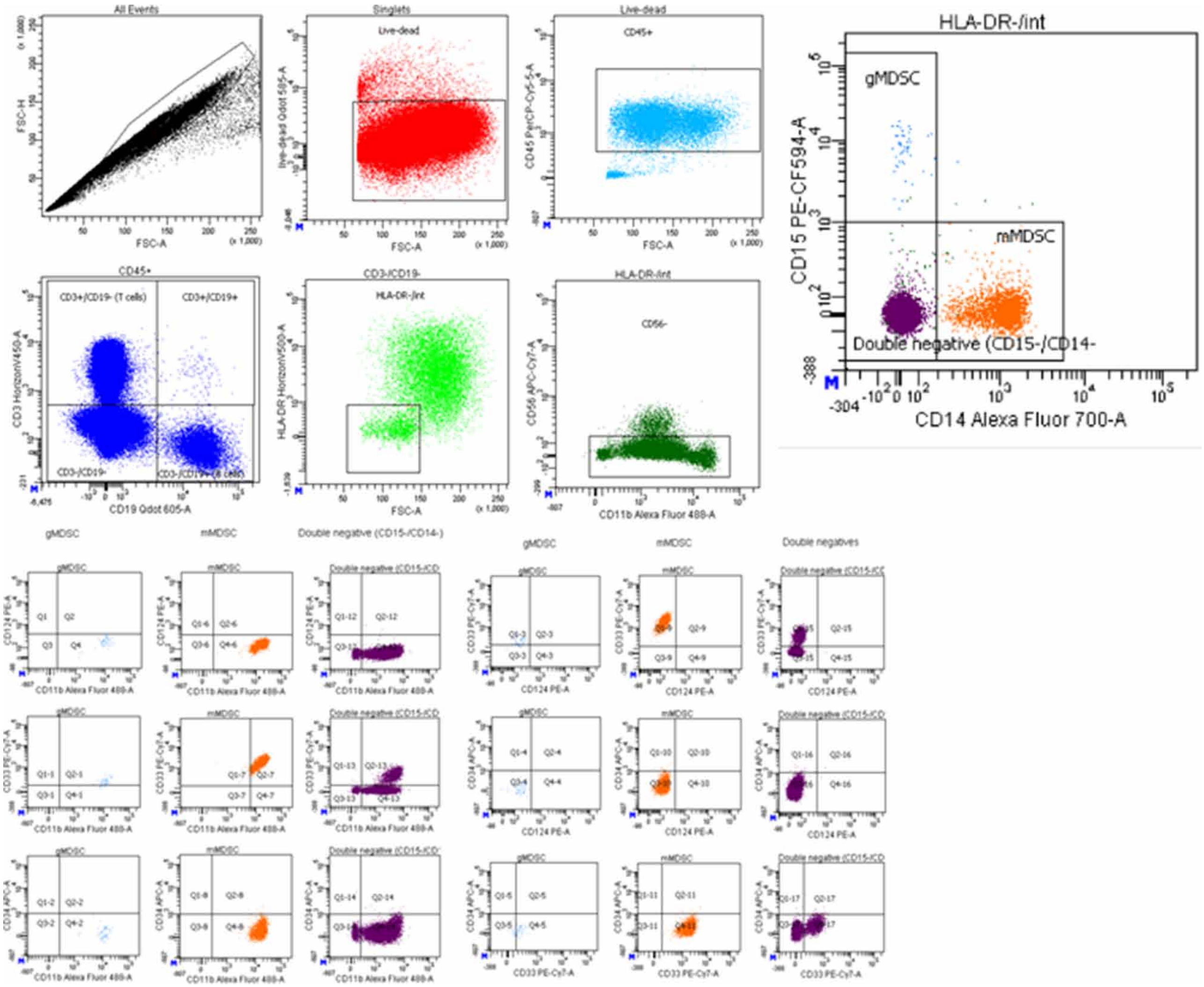

Supplementary Figure S1B: (*Continued*) Gating strategy MDSC. Singlets were gated based on FCS-A/FCS-H properties, after which the dead cells were excluded through gating on Yellow-Fluorescent Reactive Dye (Y-ARD)-negative cells. Leukocytes were gated on FSC-A/CD45. Subsequently, T-cells (CD3+CD19−), B-cells (CD3-CD19+) and myeloid cells (CD3-CD19−) were gated on differential expression of CD3 and CD19. Myeloid cells were further gated on HLA-DR$_{neg}$ cells and CD56$_{neg}$ cells. Then, three subsets were recognized: gMDSC (CD15$_{pos}$CD14$_{neg}$), mMDSC (CD15$_{neg}$CD14$_{pos}$) and double negatives (CD15$_{neg}$CD14$_{pos}$). Cells were further subdivided based on CD124, CD11b, CD33 and CD34.

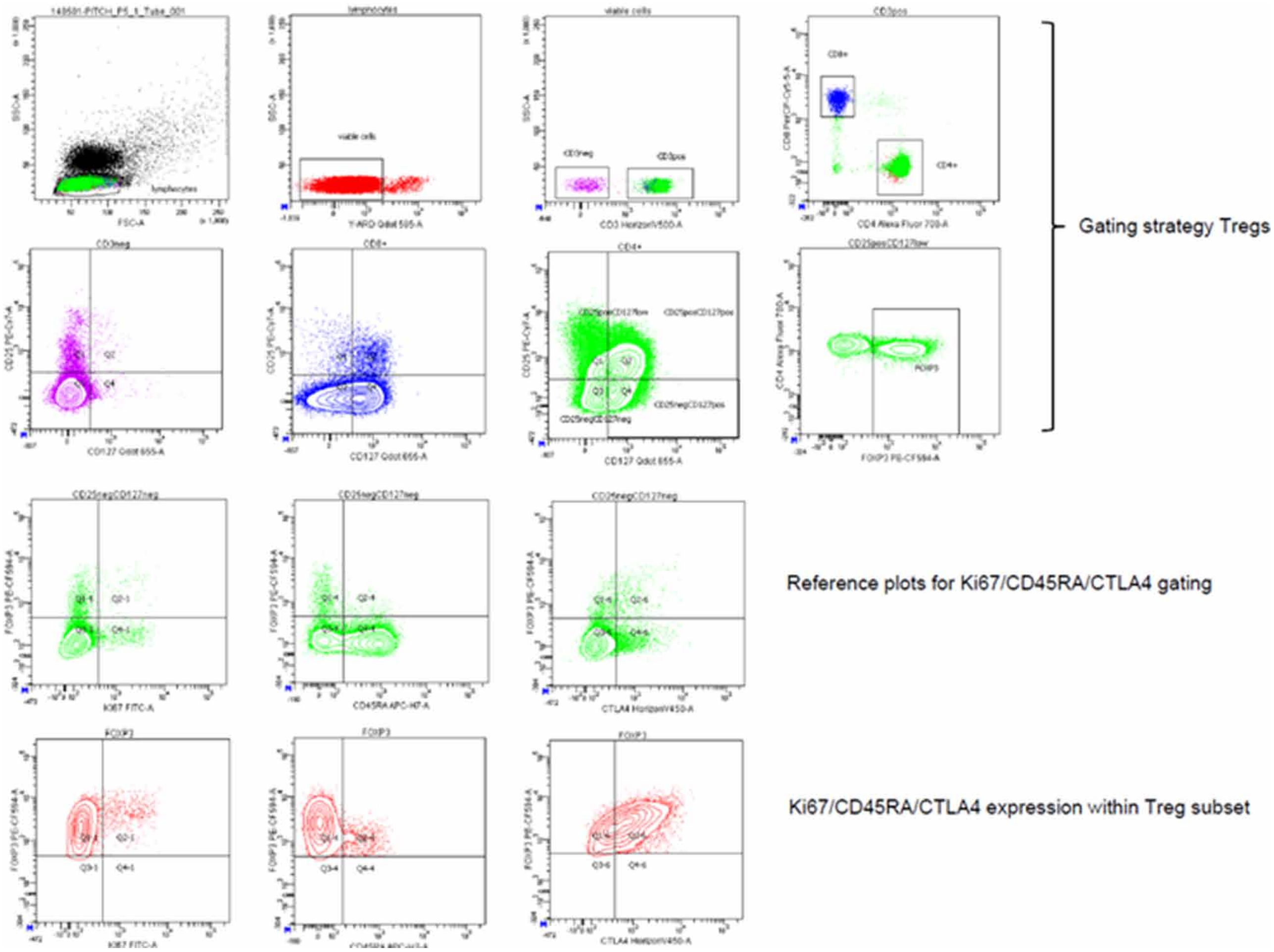

Supplementary Figure S1C: (*Continued*) Gating strategy T-regs. Lymphocytes were gated based on FSC-SSC properties, after which the dead cells were excluded through gating on Yellow-Fluorescent Reactive Dye (Y-ARD)-negative cells. Subsequently, CD3neg and CD3pos populations, as well as the CD4pos and CD8pos populations within the CD3pos cells were identified. Next, gates for CD25 and CD127 were set on CD3neg and CD8pos T cells respectively, and this gate was subsequently applied to the CD4pos T cells. FoxP3 was gated on CD25posCD127neg T cells. Gates for Ki67, CD45RA and CTLA4 were set on CD25negCD127neg T cells and applied to the CD25posCD127negFoxP3pos Tregs.

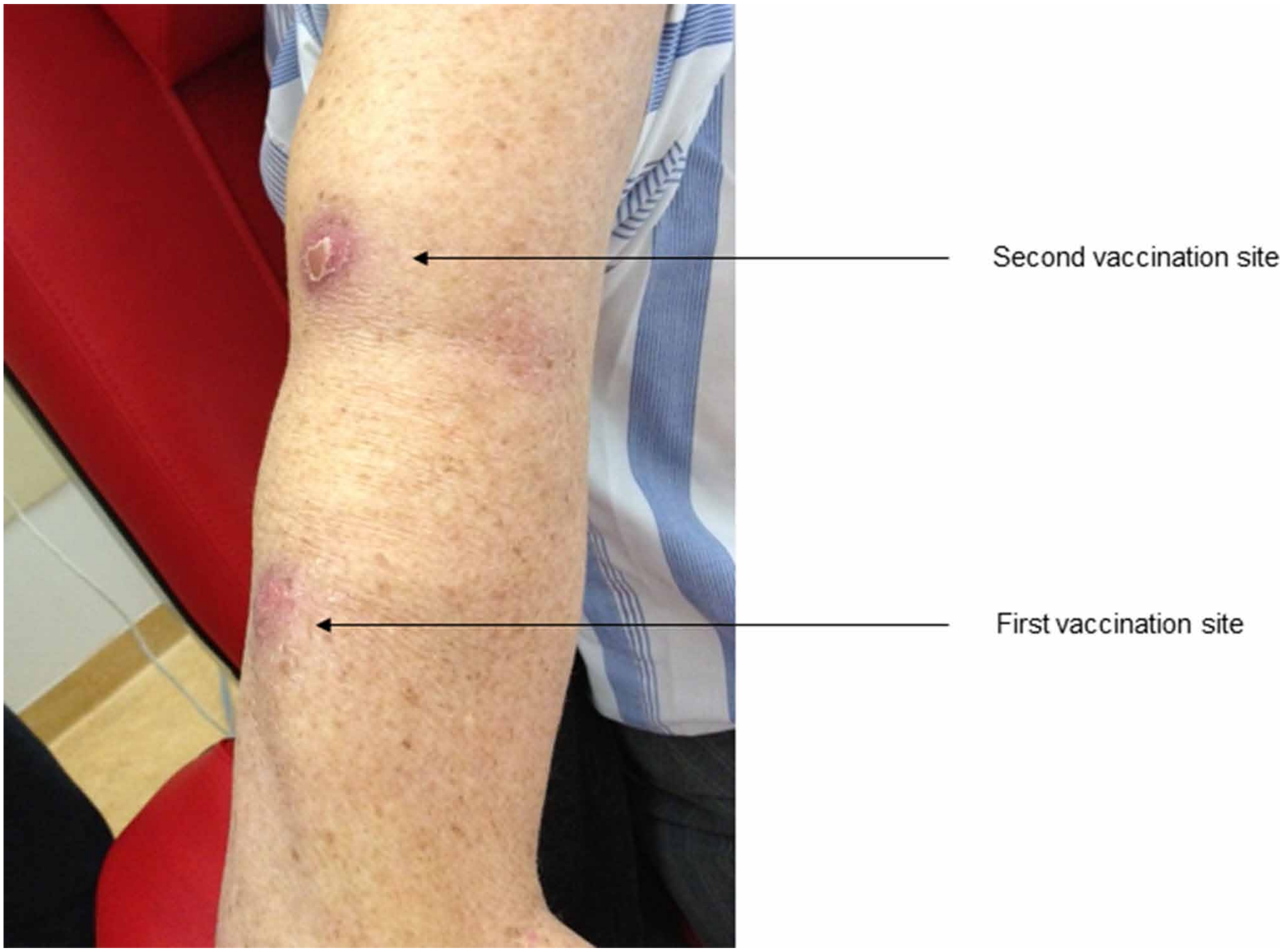

Supplementary Figure S2: Example of vaccination site of a patient. Within one week after vaccination, this patient developed induration, swelling and redness of both vaccination sides. Photo was taken two months after second vaccination.

Macrophages

CD45+ CD3- CD19- CD1a- HLA-DR+

				CD11b+CD14+ 1	CD11bintCD14int 2	CD11b-CD14- 3	CD11bintCD14- 4	CD11b-CD14int 5
CD11c+	CD163 -	CD16 -	CD206 -	6	22	38	54	70
CD11c+	CD163 +	CD16 +	CD206 +	7	23	39	55	71
CD11c+	CD163 +	CD16 -	CD206 -	8	24	40	56	72
CD11c+	CD163 +	CD16 +	CD206 -	9	25	41	57	73
CD11c+	CD163 +	CD16 -	CD206 +	10	26	42	58	74
CD11c+	CD163 -	CD16 +	CD206 +	11	27	43	59	75
CD11c+	CD163 -	CD16 -	CD206 +	12	28	44	60	76
CD11c+	CD163 -	CD16 +	CD206 -	13	29	45	61	77
CD11c-	CD163 -	CD16 -	CD206 -	14	30	46	62	78
CD11c-	CD163 +	CD16 +	CD206 +	15	31	47	63	79
CD11c-	CD163 +	CD16 -	CD206 -	16	32	48	64	80
CD11c-	CD163 +	CD16 +	CD206 -	17	33	49	65	81
CD11c-	CD163 +	CD16 -	CD206 +	18	34	50	66	82
CD11c-	CD163 -	CD16 +	CD206 +	19	35	51	67	83
CD11c-	CD163 -	CD16 -	CD206 +	20	36	52	68	84
CD11c-	CD163 -	CD16 +	CD206 -	21	37	53	69	85

MDSCs

CD45+ CD3- CD19- HLA-DR-

				CD15+CD14- 1	CD15-CD14+ 2	CD15-CD14- 3
CD11b+	CD33 -	CD34 -	CD124 -	4	20	36
CD11b+	CD33 +	CD34 +	CD124 +	5	21	37
CD11b+	CD33 +	CD34 -	CD124 -	6	22	38
CD11b+	CD33 +	CD34 +	CD124 -	7	23	39
CD11b+	CD33 +	CD34 -	CD124 +	8	24	40
CD11b+	CD33 -	CD34 +	CD124 +	9	25	41
CD11b+	CD33 -	CD34 -	CD124 +	10	26	42
CD11b+	CD33 -	CD34 +	CD124 -	11	27	43
CD11b-	CD33 -	CD34 -	CD124 -	12	28	44
CD11b-	CD33 +	CD34 +	CD124 +	13	29	45
CD11b-	CD33 +	CD34 -	CD124 -	14	30	46
CD11b-	CD33 +	CD34 +	CD124 -	15	31	47
CD11b-	CD33 +	CD34 -	CD124 +	16	32	48
CD11b-	CD33 -	CD34 +	CD124 +	17	33	49
CD11b-	CD33 -	CD34 -	CD124 +	18	34	50
CD11b-	CD33 -	CD34 +	CD124 -	19	35	51

MDSC Definitions

MDSC1	CD14+CD124+
MDSC2	CD15+CD124+
MDSC3	Lin-CD33+HLA-DR-
MDSC4	CD14+HLA-DR^{low}
MDSC5	CD15+CD14-CD11b+
MDSC6	CD15+FCS^{low}SSC^{high}
MDSC7	CD15-CD14+CD33^{hi}HLA-DR^{low}
MDSC8	CD15+CD33^{hi}
MDSC9	CD14-CD15-CD33^{hi}
MDSC10	Lin-HLA-DR^{low}CD11b+

Supplementary Figure S3: Classification of Macrophages and MDSC based on our own gating strategy. Example: MDSC # 8 is CD11b+CD14+CD11c+CD163+CD16−CD206−. Well-known MDSC definitions are listed to complete the overview.

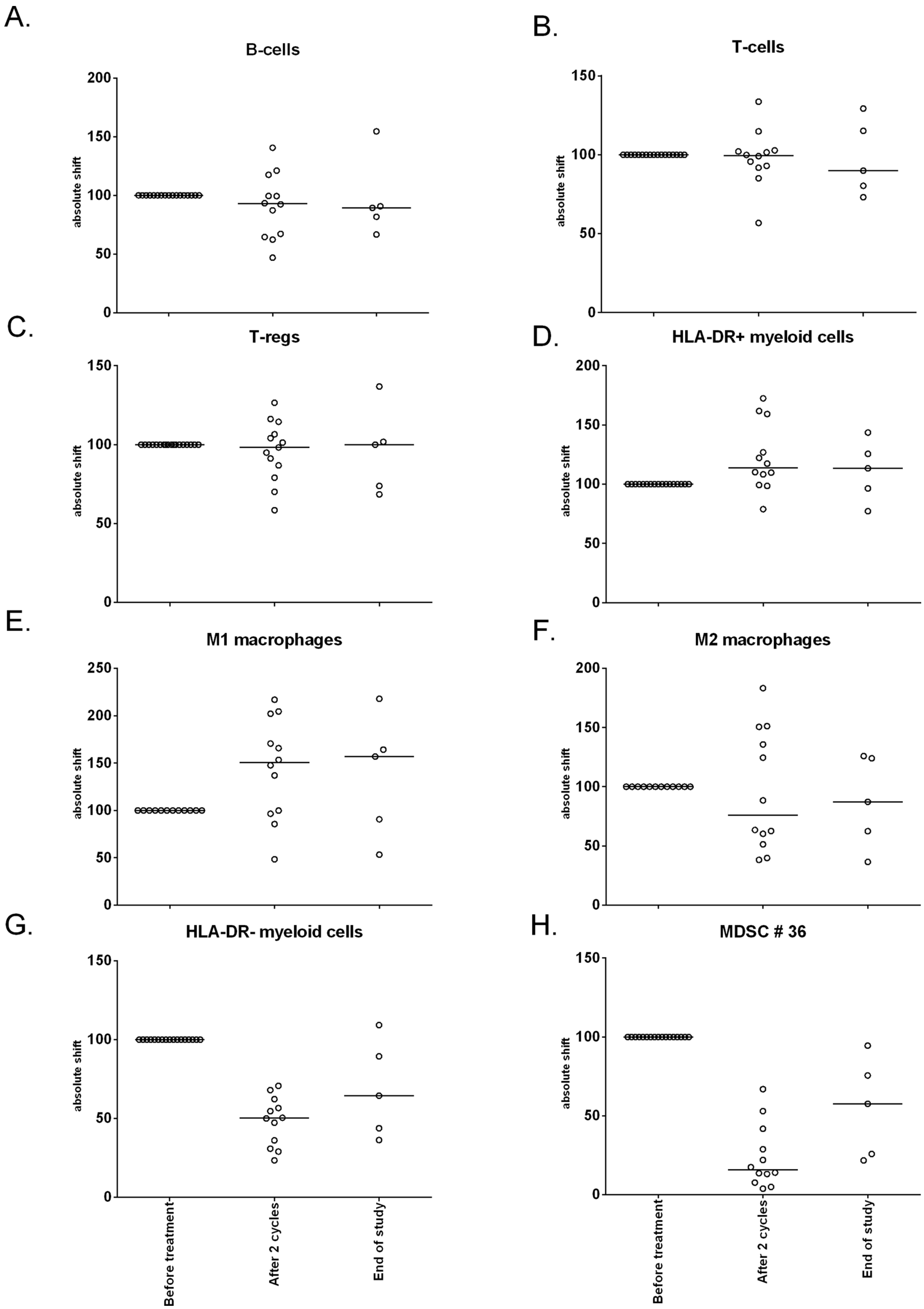

Supplementary Figure S4: Depicted here are the same figures as shown in Figure 2, but now measured as absolute shift from patients' individual measurements at baseline. Phenotypical changes upon treatment in different cell subsets, divided per treatment group. **A.** B cells **B.** T cells **C.** Tregs **D.** HLA-DR+ myeloid cells **E.** M1 macrophages **F.** M2 macrophages **G.** HLA-DR− myeloid cells MDSCs **H.** MDSC #36.

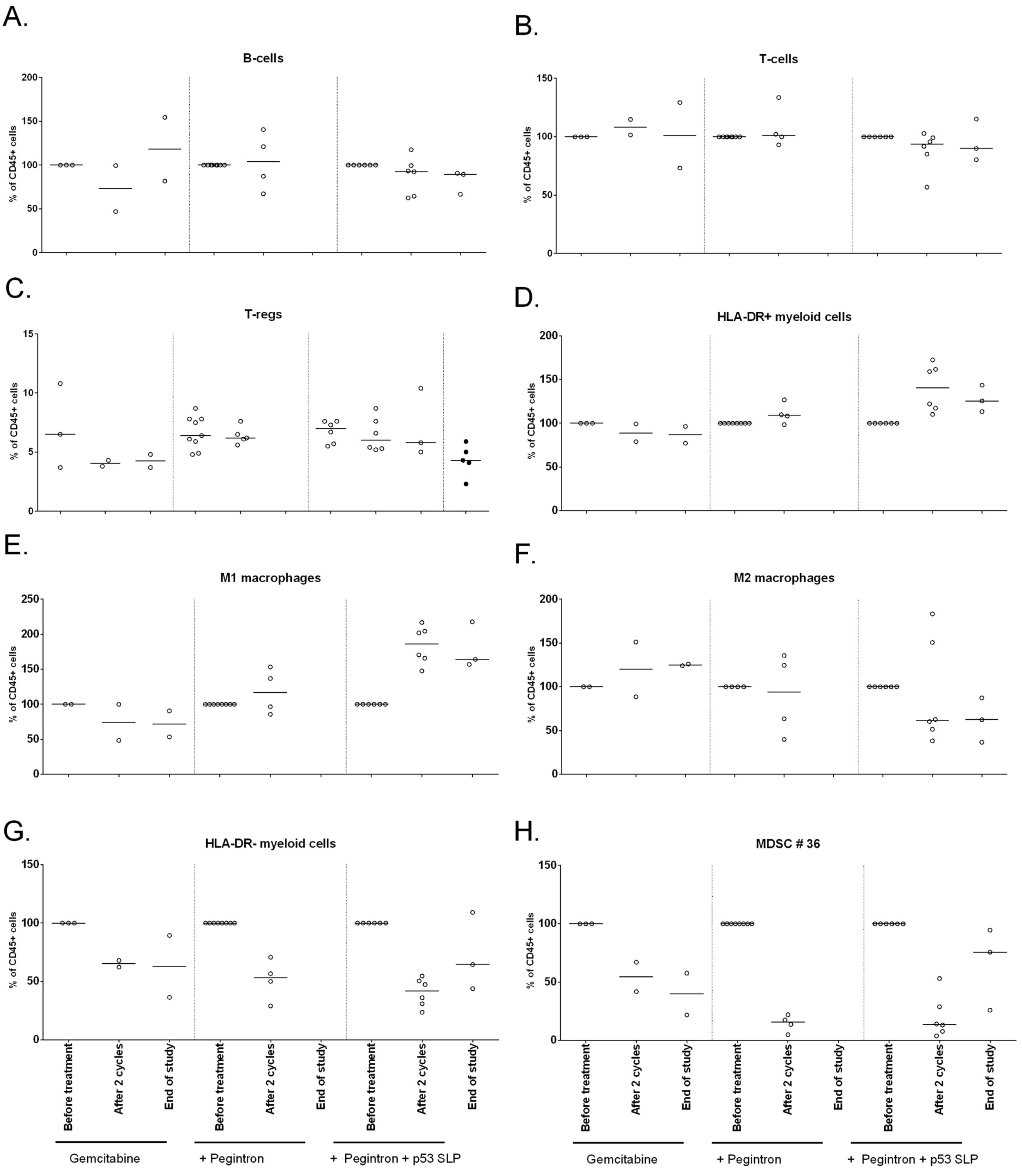

Supplementary Figure S5: Depicted here are the same figures as shown in Figure 3, but now measured as absolute shift from patients' individual measurements at baseline. Phenotypical changes upon treatment in different cell subsets, divided per treatment group. **A.** B cells **B.** T cells **C.** Tregs **D.** HLA-DR+ myeloid cells **E.** M1 macrophages **F.** M2 macrophages **G.** HLA-DR− myeloid cells **H.** MDSC #36.

p53

Before treatment — p53 peptide pools

	1	2	3	4	5	6
C01	<1	<1	<1	<1	<1	<1
C03	<1	<1	<1	<1	<1	<1
C06	<1	<1	<1	<1	<1	<1
C08	<1	<1	<1	<1	4	8
C16	<1	<1	<1	<1	<1	<1
C17	<1	<1	<1	<1	<1	<1
C18	<1	<1	<1	<1	<1	<1
C10	<1	<1	<1	<1	<1	<1
C11	<1	<1	<1	<1	5	6
C12	<1	<1	<1	<1	<1	<1
C13	<1	<1	**23**	3	<1	<1
C14	<1	<1	<1	<1	<1	<1
C15	<1	<1	<1	<1	<1	<1

After 2 cycles — p53 peptide pools

	1	2	3	4	5	6
C01	<1	<1	<1	<1	<1	<1
C03	<1	<1	3	<1	2	<1
C06	<1	<1	<1	<1	1	<1
C08	<1	**26**	**47**	8	**40**	**30**
C16	<1	<1	<1	<1	6	**14**
C17	<1	<1	<1	<1	<1	<1
C18	**18**	4	<1	3	**35**	9
C10	<1	**17**	6	**43**	**68**	2
C11	<1	**20**	**19**	**18**	**20**	4
C12	<1	**128**	**10**	**221**	**82**	5
C13	<1	**185**	**25**	**196**	**209**	<1
C14	<1	<1	**26**	**200**	**93**	<1
C15	<1	<1	**23**	<1	**13**	<1

Last visit — p53 peptide pools

	1	2	3	4	5	6
C01	na	na	na	na	na	na
C03	<1	<1	<1	<1	1	<1
C06	na	na	na	na	na	na
C08	na	na	na	na	na	na
C16	na	na	na	na	na	na
C17	<1	<1	<1	2	<1	<1
C18	na	na	na	na	na	na
C10	<1	<1	3	**10**	**21**	<1
C11	na	na	na	na	na	na
C12	na	na	na	na	na	na
C13	**54**	**227**	**41**	**214**	**209**	<1
C14	<1	<1	<1	**15**	9	<1
C15	na	na	na	na	na	na

FLU / MRM

Before treatment — Flu peptide pools / MRM

	1	2	3	4	MRM
C01	<1	5	2	8	**22**
C03	**84**	**15**	**19**	3	**52**
C06	<1	3	1	<1	2
C08	**18**	**53**	**23**	**33**	**23**
C16	<1	**79**	<1	**37**	<1
C17	na	na	na	na	**62**
C18	<1	<1	<1	<1	**31**
C10	**22**	3	3	7	7
C11	5	**19**	**26**	6	7
C12	8	7	<1	<1	**53**
C13	**124**	**90**	<1	**21**	**199**
C14	**45**	**54**	<1	6	**119**
C15	<1	<1	<1	<1	<1

After 2 cycles — Flu peptide pools / MRM

	1	2	3	4	MRM
C01	2	**24**	3	**16**	**17**
C03	**44**	**48**	**26**	9	**12**
C06	<1	5	**17**	**22**	**18**
C08	**58**	**79**	**44**	**38**	**199**
C16	**59**	**156**	**16**	**119**	**13**
C17	2	<1	2	7	**22**
C18	<1	4	<1	66	1
C10	**16**	**21**	**12**	**15**	**13**
C11	**14**	**25**	9	**15**	**19**
C12	**19**	3	8	<1	**135**
C13	**159**	**94**	**16**	**12**	**60**
C14	**14**	**114**	**13**	**14**	**229**
C15	<1	<1	<1	<1	<1

Last visit — Flu peptide pools / MRM

	1	2	3	4	MRM
C01	na	na	na	na	na
C03	**43**	**27**	**16**	<1	**44**
C06	na	na	na	na	na
C08	na	na	na	na	na
C16	na	na	na	na	na
C17	<1	<1	<1	<1	**32**
C18	na	na	na	na	na
C10	**10**	2	<1	4	3
C11	na	na	na	na	na
C12	na	na	na	na	na
C13	**81**	**97**	<1	**38**	**123**
C14	5	**35**	<1	9	**234**
C15	na	na	na	na	na

na: not available

- Gemcitabine (white: C01, C03)
- Gemcitabine + IFN-α (grey: C06, C08, C16, C17, C18)
- Gemcitabine + IFN-α + p53 SLP ® (black: C10, C11, C12, C13, C14, C15)

Supplementary Figure S6: Cellular immune responses to the vaccine were assessed by an IFN-γ ELISPOT assay. Here depicted are ELISPOT results per peptide pool. **Bold** marks a positive response; avaccine-induced response was defined as at least a 3-fold increase in response after vaccination compared with the baseline sample. The top 3 boxes represents the response against the p53 peptide pools (1-6 are described in materials and methods). Bottom 3 boxes show the results against Flu peptides and MRM.

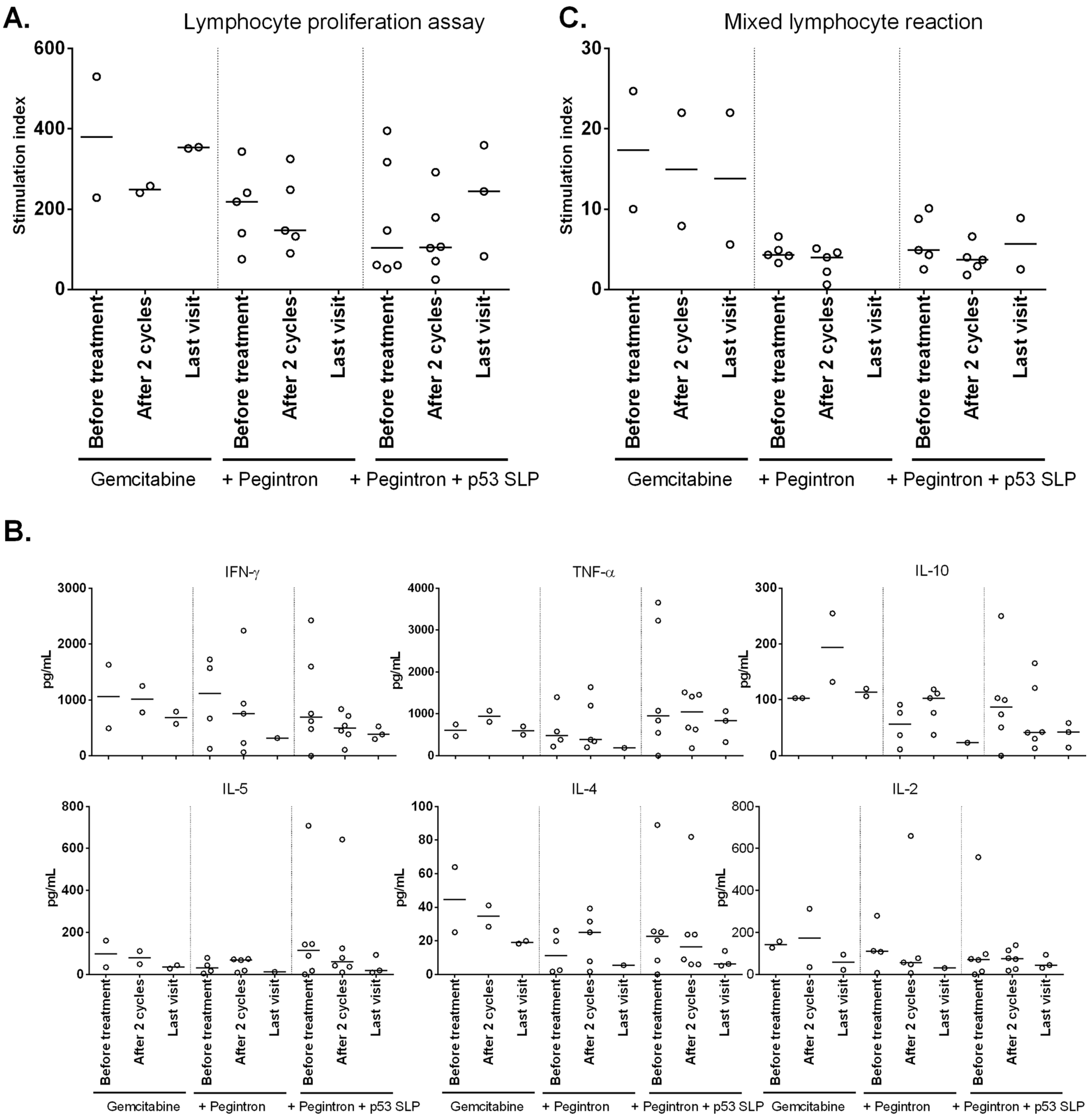

Supplementary Figure S7: A. Outcomes of lymphocyte proliferation assay; cells were stimulated with PHA **B.** Outcomes of mixed lymphocyte reaction **C.** Cytokine production of IFN-a, TNF-a, IL-10, IL-5, IL-4 and IL-2 measured in lymphocyte proliferation assay as desribed under A. Cytokines were measured 48 hours after stimulation. There were no changes in antigen presenting capacity. Patients display a more pronounced Th1 profile of secreted cytokines**.**
